# Soil microbial sensitivity to temperature remains unchanged despite community compositional shifts along geothermal gradients

**DOI:** 10.1111/gcb.15878

**Published:** 2021-09-28

**Authors:** Gabriel Y. K. Moinet, Manpreet K. Dhami, John E. Hunt, Anastasija Podolyan, Liyĭn L. Liáng, Louis A. Schipper, David Whitehead, Jonathan Nuñez, Adriano Nascente, Peter Millard

**Affiliations:** ^1^ Soil Biology Group Wageningen University and Research Wageningen The Netherlands; ^2^ Manaaki Whenua – Landcare Research Lincoln New Zealand; ^3^ Manaaki Whenua – Landcare Research Palmerston North New Zealand; ^4^ University of Waikato Hamilton New Zealand; ^5^ Embrapa Arroz e Feijão Santo Antônio de Goiás Goiás Brazil

**Keywords:** geothermal warming, macromolecular rate theory, microbial community composition, microbial thermal adaptation, soil carbon, soil organic matter decomposition, temperature sensitivity

## Abstract

Climate warming may be exacerbated if rising temperatures stimulate losses of soil carbon to the atmosphere. The direction and magnitude of this carbon‐climate feedback are uncertain, largely due to lack of knowledge of the thermal adaptation of the physiology and composition of soil microbial communities. Here, we applied the macromolecular rate theory (MMRT) to describe the temperature response of the microbial decomposition of soil organic matter (SOM) in a natural long‐term warming experiment in a geothermally active area in New Zealand. Our objective was to test whether microbial communities adapt to long‐term warming with a shift in their composition and their temperature response that are consistent with evolutionary theory of trade‐offs between enzyme structure and function. We characterized the microbial community composition (using metabarcoding) and the temperature response of microbial decomposition of SOM (using MMRT) of soils sampled along transects of increasing distance from a geothermally active zone comprising two biomes (a shrubland and a grassland) and sampled at two depths (0–50 and 50–100 mm), such that ambient soil temperature and soil carbon concentration varied widely and independently. We found that the different environments were hosting microbial communities with distinct compositions, with thermophile and thermotolerant genera increasing in relative abundance with increasing ambient temperature. However, the ambient temperature had no detectable influence on the MMRT parameters or the relative temperature sensitivity of decomposition (*Q*_10_). MMRT parameters were, however, strongly correlated with soil carbon concentration and carbon:nitrogen ratio. Our findings suggest that, while long‐term warming selects for warm‐adapted taxa, substrate quality and quantity exert a stronger influence than temperature in selecting for distinct thermal traits. The results have major implications for our understanding of the role of soil microbial processes in the long‐term effects of climate warming on soil carbon dynamics and will help increase confidence in carbon‐climate feedback projections.

## Graphical abstract

Soils samples along geothermal gradients (at 2, 10 and 30 m from a geothermally active zone) of increasing mean annual temperature (MAT) in two biomes (kanuka stand and grassland) presented shifts in the composition of microbial communities (as shown by the non‐parametric multidimensional scaling (MDS) plots), but similar relative temperature sensitivities (*Q*_10_). The temperature sensitivity was however correlated with the carbon content of soil samples, which varied independently from temperature. The results suggest that while long‐term warming selects for warm‐adapted taxa, substrate quantity exerts a stronger influence than temperature in selecting for distinct thermal traits.

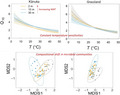

## 1 INTRODUCTION

Microbial decomposition of soil organic matter (SOM) results in emissions of up to 60 Pg (10^15^ g) of carbon (C) per year as CO_2_ from soils to the atmosphere (Cavicchioli et al., 2019; Reay, 2007)—approximately six times the current annual rate of anthropogenic emissions—making it a key component in the global C cycle. Yet, large knowledge gaps remain that feed uncertainties around the temperature sensitivity of soil microbial processes, thereby limiting the confidence in Earth System Models projections (Bradford et al., 2016). Indeed, despite hundreds of studies on the topic, it remains unknown whether increasing temperatures will result in global soil C stocks losses and enhance the rate of global warming via a self‐reinforcing positive feedback loop, or on the contrary, whether it will result in global soil C increases leading to self‐attenuating negative feedback loop (Bradford et al., 2016; van Gestel et al., 2018).

Evidence from short‐term studies of the exponential increase of microbial decomposition of SOM with temperature is used in models that predict the rate of increase in atmospheric CO_2_ concentration and the consequent effects on climate change (Friedlingstein et al., 2006; Kirschbaum, 2006). However, this increase can be short‐lived, with decomposition rates often, but not always (Carey et al., 2016; Hartley et al., 2008) returning to pre‐warming levels when warming is sustained for several years (Luo et al., 2001; Melillo et al., 2002, 2017). Understanding the mechanisms underlying the temporal dynamics of the temperature response of SOM decomposition is critical for improving predictions of future climate, but they remain the subject of scientific debate.

Two non‐mutually exclusive mechanisms have been proposed to explain shifts in microbial decomposition of SOM rates over time during sustained warming: (1) microbial thermal adaptation (thereafter simply referred to as thermal adaptation; Bradford, 2013), that is, a shift in the physiology of microbial decomposers observable at the community scale leading to a decrease in the relative temperature sensitivity of decomposition (the proportional change in decomposition rate per unit temperature) and (2) substrate depletion (Kirschbaum, 2004). It is well established that substrate depletion can attenuate the response of microbial decomposition to temperature by increasing constraints on microbial access to substrates (Davidson & Janssens, 2006; Kirschbaum, 2013; Moinet et al., 2018, 2020). In contrast, the role of thermal adaptation in explaining temporal dynamics of SOM decomposition undergoing long‐term warming remains unknown. Thermal adaptation is supported theoretically by the evolutionary trade‐offs between enzyme structure and function such that enzymes benefit from a rigid structure (inflexible) at higher temperature to increase substrate–enzyme affinity (Bradford, 2013). Therefore, warming should select for microbial communities with relatively high proportions of microbial taxa that produce warm‐adapted, rigid enzymes, counteracting the positive effect of increasing temperature on reaction rates (Bradford, 2013; Hochachka & Somero, 2002). This has recently been identified as the ‘enzyme rigidity hypothesis’ (Alster et al., 2020). However, empirical evidence does not point to a consistent conclusion. Indeed, recent observations supporting thermal adaptation in conditions of unlimited substrate supply (Bradford et al., 2019; Dacal et al., 2019) contrast with earlier observations of increased sensitivity (Auffret et al., 2016; Karhu et al., 2014) or no change in sensitivity (Walker et al., 2018) during long‐term warming.

Most studies that have attempted to describe the temperature response of soil microbial decomposition have used the Arrhenius model, or empirical modifications of it that improve the fit to SOM decomposition data (Alster et al., 2020). It is increasingly clear that these models are not well‐suited to describe the microbial decomposition of SOM (and other biological processes), for several well‐documented reasons (Davidson & Janssens, 2006; Schipper et al., 2014; Tang & Riley, 2015). For example, a major constraint of these models is that they do not include a maximum or inflection point beyond which respiration rates decline. More recently, the macromolecular rate theory (MMRT) has been successfully applied to a range of soil microbial processes (Alster et al., 2018; Numa et al., 2021; Robinson et al., 2020; Schipper et al., 2014, 2019) and is known to account for the limitations of the Arrhenius‐derived models by incorporating enzymatic behaviour using thermodynamics (Hobbs et al., 2013; Prentice et al., 2020). Parameters derived from MMRT at the microbial community scale reflect the sum of activities of individual microbes and enzymes and can be considered as traits of microbial communities (Alster et al., 2016, 2018). MMRT, therefore, offers a unique opportunity to address hypotheses related to thermal adaptation using intrinsic characteristics of the microbial community (Alster et al., 2020). In particular, shifts in *T*_opt_, the optimum temperature at which decomposition rates are highest and $\Delta C_{P}^{‡}$, the change in heat capacity that regulates the steepness of the temperature response, were recently hypothesized to explain long‐term trends in the temperature response in line with enzymatic theory (Alster et al., 2020).

In this study, we apply MMRT to describe the temperature response of microbial decomposition in a natural long‐term warming experiment using measurements along a gradient in a geothermally active area of the Taupō Volcanic Zone, New Zealand. Our objective was to test whether microbial communities adapt to long‐term warming with a shift in their temperature response consistent with the ‘enzyme rigidity hypothesis’. The choice of natural geothermal gradients as an experimental setup allowed us to study the effect of long‐term warming, in the field, with a minimum of confounding factors (O'Gorman et al., 2014).

We sampled soils along transects in three locations of increasing distance from a geothermally active area defining a thermal environment treatment. We established the transects in two biomes (a shrubland and a grassland) and soils were sampled at two depths to ensure the temperature varied independently of soil C concentration. The sampling strategy resulted in a full factorial design (3 thermal environments × 2 biomes × 2 depths), replicated three times, allowing us to disentangle the effects of substrate supply (as approximated from C concentration) from those of long‐term warming on the temperature response of microbial decomposition of SOM. For all treatment, metabarcoding was used to characterize the composition of microbial communities. Our specific objectives were to test the following hypotheses: (i) warming selects for microbial communities with distinct composition characterized by increasing relative abundance of thermophile and thermotolerant organisms and (ii) these communities have adapted to their environmental temperature in such a way that they have a higher *T*_opt_ and a flatter temperature response (less negative $\Delta C_{P}^{‡}$), in accordance with the ‘enzyme rigidity hypothesis’ (Alster et al., 2020; Arcus et al., 2016), leading to a lower relative temperature sensitivity.

## 2 MATERIALS AND METHODS

### 2.1 Site description

The study site was located east of the town of Taupō (38°41.340ʹS, 176°7.151ʹE, 475 m a.s.l.), within the Wairakei‐Tauhara geothermal field, which is part of the geothermally active Taupō Volcanic Zone, North Island, New Zealand. The local geothermal surface features consist of a discontinuous ring of steam‐heated ground, with steam vents and fumaroles in bare soil depressions, each about 10 m wide, 2 m deep and 80–200 m long, enclosing an area of about 80 ha. Hereafter, these geothermal features are referred to as heated depressions. These features are at least 1820 years old (Cody, 2007).

The immature orthic pumice sandy‐loam soil (Typic Udivitrand) is deep and well drained with a very shallow A horizon. The main vegetation is grazed grassland dominated by grasses of the genus *Paspalum* (Poaceae). However, the edges of the steam‐heated depressions are often dominated by prostate kānuka, *Kunzea tenuicaulis* de Lange (Myrtaceae), an endemic shrub up to 2 m tall, with a natural range restricted to the geothermally active areas in the Central Volcanic Field (North Island) and it forms a well‐constrained, microhabitat zone of 5–25 m wide (Nishar et al., 2017).

### 2.2 Experimental design

In early August 2018, transects were established at six thermal gradients perpendicular to the steam‐heated depressions. Three transects were dominated by kānuka and three by grassland ecosystems. Three distances along each thermal gradient, near the heat source (ca. 2 m from the heated depression), at an intermediate distance (ca. 10 m) and in an area assumed to be unaffected by ground heating (ca. 30 m) defined three thermal environment treatments, hereafter referred to by their distance to the heated depressions (2, 10 and 30 m). At each sample point, two temperature sensors with dataloggers were installed into the soil, one at a depth of 50 mm and measuring at 2 hourly intervals (Hobo MX2201; Onset Computer Corp.) and one at a depth of 100 mm and measuring at 6 hourly intervals (Thermochron iButton DS1921G; Maxim Integrated). This setup defined 36 sampling points following a full factorial design across three replicates (transects) with three distances from the heated depression (defining different thermal environments; 2, 10 and 30 m), two biomes and two depths (Figure S1).

All dataloggers were collected 4 months after installation following soil sampling, and the data were used to characterize the thermal environment at each sampling point. The average soil temperature over the 4 months of measurements at each sampling point is referred to as mean environmental temperature (MET).

### 2.3 Soil sampling

In November 2018 (early spring), approximately 500 g of bulk soil was collected from each of the 36 sampling points for soil respiration measurements, placed in a sealed bag and stored in an insulated container until it was placed in a refrigerator at 4°C at the end of each sampling day. Three additional cores were sampled at each sampling point to characterize microbial (bacterial and fungal) communities. These samples were collected using sterilized PVC cylinder cores (50 mm diameter, 150 mm height) hammered into the soil to a depth of 120 mm. After collection, the cylinder was cut open length‐wise in the field using a fine Dremel^®^ saw and surface soil was removed using a sterile spatula. Soil was sampled at 0–50 and 50–100 mm depths, representing two distinct horizons comprising the dark coloured humus‐rich top soil and the subsurface mineral‐rich fraction. Then, 0.5 g of each sample was transferred to sterile tubes (MN Bead Tubes Type A from the MN Nucleospin 96 soil kit [MN740787.4; Machery Nagel GmbH & Co.]) and kept on ice in the field, followed by storage at −20°C until DNA extraction was undertaken.

Soil volumetric water content (*W*_s_) was measured at each location using a portable sensor (ML3; Delta‐T Devices Ltd.) on each day of sampling.

### 2.4 Laboratory measurements of soil respiration

All soils were taken to the laboratory and the fresh samples were sieved at 2 mm and stored at 4°C for 10 days, after which measurements of soil respiration rates (*R*_s_; a proxy for microbial decomposition of SOM) were made. A subsample was taken for measurement of carbon (C) and nitrogen (N) concentration using a CN analyser (Model TruMAc; LECO Corporation). pH was measured after the soil samples were shaken in distilled water (2 soil:5 water mass/volume).

Each soil sample was subdivided into 22 subsamples placed in separate septum‐sealed 12 ml Exetainer^®^ vials (Labco) for measurements of *R*_s_ at 22 different incubation temperatures (approximately 4–48°C with 2°C increments). The exetainers containing the soil samples were placed in temperature‐controlled polystyrene boxes (36 vials per box). Two fine thermocouples (Type T; Omega Engineering Ltd.) were installed in each box, one in a mock vial with spare soil from the field sampling site, and one attached to a heating pad placed at the bottom of the box. The control of the box temperature (±0.5°C) to a predetermined setpoint was carried out using a datalogger (CR1000; Campbell Scientific). The datalogger also recorded the average temperature of the soil samples at 15 min intervals. Boxes with target incubation temperatures lower than 25°C were placed in a growth chamber with an ambient temperature adjusted to within 1°C of the box target temperature. Boxes with target temperatures higher than 25°C were kept in the laboratory, except for the boxes with target temperatures of 46 and 48°C, which were placed in an oven with temperature set at 40°C.

For measurements of *R*_s,_ all samples were placed in their respective temperature‐controlled boxes for approximately 2 h, until soil temperatures reached their target values. Measurements of *R*_s_ were then made sequentially for each box (all samples in each box at the same time) by removing the exetainers, sealing the vial by closing the rubber septum and then measuring the CO_2_ concentration in the headspace at time 0. The exetainers were then replaced in the box at the target temperature, and the soils incubated for a sufficient time to produce a measurable change in headspace CO_2_. Finally, headspace CO_2_ concentrations were measured again. The CO_2_ concentration was determined by injection of 0.5 ml of headspace gas into a continuous flow of CO_2_‐free air passing through a calibrated infra‐red gas analyser (LI‐7500; LI‐COR).

Samples held at lower temperatures needed longer incubation times than those at warmer temperatures to produce a similar sufficient increase in CO_2_ concentration. To minimize differences in the incubation times, we varied the mass of the soil subsample such that 4 g (equivalent dry mass) was used for target incubation temperatures lower than 20°C, 3 g for target temperatures of 20–30°C, 2 g for target temperatures of 30–38°C and 1 g for target temperatures higher than 38°C. Robinson et al. (2017) observed linear increase in CO_2_ concentrations for soil incubations up to 6 h. The incubation times in our study varied between 52 and 169 min and we assumed linearity for all measurements. All 1584 measurements of CO_2_ concentrations were made within 1 day. Values of *R*_s_ were calculated by subtracting the initial CO_2_ concentration in the tube from the concentration at the end of the incubation, using the vial gas volume to convert from measured CO_2_ concentration to moles of CO_2_ and dividing by the mass of oven‐dried soil and the incubation time.

### 2.5 DNA extraction, PCR amplification and amplicon sequencing

Total genomic DNA was extracted from soil samples using the MN kit nucleospin soil 96 kit as per the manufacturer's instructions with the following modifications. Soil was mixed with the SL1 buffer and SX enhancer and incubated in an orbital mixer incubator (Ratek) at +65°C, 4 *g* for 20 min. Samples were homogenized for 2 × 10 min at 30 Hz in a TissueLyser II Beadmill (Qiagen). The remaining DNA extraction steps were performed on a Janus^®^ G3 MDT (PerkinElmer) robotic workstation, with the resulting DNA suspended in 100 µl of SE buffer and then stored at −20°C. DNA quality and quantity were assessed with a spectrometer (NanoDrop 2000; Thermo Fisher Scientific) to ensure successful extraction.

To assess microbial community identity and structure, we amplified the hyper‐variable V4 region of the bacterial 16S rRNA gene (515f/806r primer pair; Caporaso et al., 2011) and the fungal ITS1 gene (ITS1‐KYOF/ITS2‐KYO2 primer pair; Toju et al., 2012), as described in Toju et al. (2018). We coamplified both 16S and ITS1 regions in a duplex polymerase chain reaction (PCR) (*T*_*anneal*_ = 50°C), using 400 nM of each primer pair in a Kapa3G Plant PCR kit that contains High‐Fidelity Taq polymerase (Kapa Biosystems). For each amplification, we employed the Nex‐F fusion primer strategy, which included 3–6 mer N‐spacers to increase base‐diversity (Lundberg et al., 2013), linker sequences to attach forward and reverse 8‐mer dual index tags and illumina sequencing adaptor addition in a second amplification step. In addition to soil samples, extraction and PCR negative controls, and synthetic Synmock communities (Palmer et al., 2018) were included as controls in library formation. All amplified products were normalized, purified and size‐selected using Sera‐Mag speed beads (Sigma‐Aldrich) as described in Dhami et al. (2018), quantified using Qubit (dsDNA HS Assay Kit; Invitrogen) and pooled equimolar to form amplicon libraries. The libraries were assessed for amplicon size distribution and quantified using a LabChip^®^ GX Touch^TM^ Nucleic Acid Analyzer (PerkinElmer) and Qubit® 2.0 Fluorometer (Invitrogen), diluted to 4 nM, and sequenced using a 10% PhiX spike‐in and the Illumina dye sequencing technique on a MiSeq 3000 system (Illumnia Inc.) at the University of Auckland Genomics Facility (2 × 250 cycle sequencing kit).

### 2.6 Soil respiration responses to temperature

Soil respiration at the different temperatures was fitted using MMRT (Equation 1) for each of 35 sampling points (one of the soil samples from the kānuka biome did not release any CO_2_ from any of the incubation temperatures and was discarded from the analysis):

(1) $$\ln\left(R_{s}\right)=\ln\left[\frac{k_{B}T}{h}\right]‐\frac{\Delta H_{T_{0}}^{‡}+\Delta C_{P}^{‡}\left(T‐T_{0}\right)}{\mathrm{RT}}+\frac{\Delta S_{T_{0}}^{‡}+\Delta C_{P}^{‡}\left(\ln(T)‐\ln(T)\right)}{R},$$

where *R* is the universal gas constant, *T* is the measurement temperature in K and *T*_0_ is a reference temperature (25°C, 298 K), *h* is Planck's constant, and *k*_B_ is Boltzmann's constant. The three parameters are $\Delta C_{P}^{‡}$, the change in heat capacity between the enzyme–substrate complex and the enzyme transition state complex; $\Delta H_{T_{0}}^{‡}$, the change in enthalpy; and $\Delta S_{T_{0}}^{‡}$ the change in entropy between the enzyme–substrate complex and the enzyme transition state complex at *T*_0_.

From these parameters, we calculated the temperature optimum (*T*_opt_; Equation 2) and the temperature at which the sensitivity of soil respiration was greatest (*T*_inf_, the inflection point; Equation 3). In addition, we estimated the rate of soil respiration at the reference temperature 25°C (*R*_25_) following Liáng et al. (2018) (Equation 4).

(2) $$T_{\text{opt}}=\frac{\Delta H_{T_{0}}^{‡}‐\Delta C_{P}^{‡}T_{0}}{‐\Delta C_{P}^{‡}‐R},$$

(3) $$T_{\inf}=\frac{\Delta H_{T_{0}}^{‡}‐\Delta C_{P}^{‡}T_{0}}{‐\Delta C_{P}^{‡}+\sqrt{‐\Delta C_{P}^{‡}R}},$$

(4) $$R_{25}=\exp\left(\ln\left[\frac{k_{B}T_{0}}{h}\right]‐\frac{\Delta G_{T_{0}}^{‡}}{RT_{0}}\right),$$

where $\Delta G_{T_{0}}^{‡}=\Delta H_{T_{0}}^{‡}‐T_{0}\Delta S_{T_{0}}^{‡}$.

We also fitted the temperature response of *R* using the Lloyd and Taylor (1994) equation.

(5) $$R=R_{10}\exp\left(E_{0}\left(\frac{1}{56.02}‐\frac{1}{T‐227.1}\right)\right),$$

where *R*_10_ is a basal respiration rate at 10°C, and *E*_0_ is related to the temperature sensitivity of the decomposition of SOM.

The temperature sensitivity can be calculated in absolute terms (the absolute amount of change in the decomposition rate per unit change in temperature) as the first derivative of the model describing *R*_s_ relative to temperature (*dR*_s_/*dT*). Alternatively, the sensitivity can be calculated in relative terms (the proportional change in decomposition rate per unit temperature) as the first derivative relative to temperature divided by the reaction rate (*dR*_s_/*R*_s_ *dT*; Sierra, 2012). The relative sensitivity is therefore relative to the decomposition rate itself and is not influenced by the size of the substrate pool (the total amount of carbon) while the absolute temperature sensitivity does depend on the substrate pool size (Sierra, 2012). It is important to emphasize that only measures of the relative temperature sensitivity can be used to interpret changes specific to the physiology of the microbial community.

To compare estimates of relative temperature sensitivities from both models, we derived values of *Q*_10_ for each of the 35 curves from both MMRT following Schipper et al. (2014) (Equation 6) and Lloyd & Taylor following Moinet & Millard (2020); Equation 7). *Q*_10_ is by far the most common estimator of relative temperature sensitivity, and we preferred this to the estimator used by Sierra (2012) (*dR*_s_/*R*_s_ *dT*) for comparison with other studies.

(6) $$Q_{10}=\exp\left(\frac{10\left(\Delta H^{‡}‐5\Delta C_{P}^{‡}\right)}{RT^{2}}\right),$$

where $\Delta H^{‡}=\Delta H_{T_{0}}^{‡}+\Delta C_{P}^{‡}\left(T‐T_{0}\right)$.

(7) $$Q_{10}=\exp\left(\frac{10E_{0}}{{\left(T‐227.1\right)}^{2}}\right).$$

### 2.7 Statistical analysis

All analyses were conducted in R version 3.4.2 (R Core Team, 2017).

We assessed and compared performances of the MMRT and Lloyd & Taylor models to describe the data by calculating and comparing their respective corrected Akaike's information criterion (AIC_c_), the model with lower AIC_c_ value being a better fit. Since MMRT provided a better fit in all cases, further analyses were performed mainly on MMRT parameters.

We then conducted two further sets of analyses. The first set aimed at describing the differences in environmental variables (MET, *W*_s_, C concentration, N concentration, C:N ratio, and pH), estimated MMRT parameters ($\Delta H_{T_{0}}^{‡}$, $\Delta C_{P}^{‡}$, *T*_opt_, *T*_inf_ and *R*_25_) and microbial community composition across the combination of the three treatments depth, biome and distance from the heated depression. We assessed the effects of the different treatments on environmental variables and MMRT parameters using three‐way ANOVA. The statistical analyses used to assess differences in microbial community composition between the treatments are described in the following section.

The second set of analyses aimed at describing the influence of environmental variables on the MMRT parameters across treatments. The treatments resulted in a set of environmental conditions in which environmental temperature (MET), *W*_s_ and C concentration varied independently, but in which C:N ratio, N concentration and C concentration were strongly positively correlated between them, and pH was strongly negatively correlated with MET. We assessed the effects of environmental variables on the estimated MMRT parameters, as well as Lloyd & Taylor's parameter *E*_0_, using a backwards stepwise regression approach (Zuur et al., 2009). For each parameter separately, the estimate from the fits for each of the 35 curves was treated as a sample. Due to the correlations between variables described above, pH, N concentration and C:N were excluded from the full model. The full linear models included only the triple‐way interaction between MET, *W*_s_ and C concentration. Non‐significant interactions and variables were dropped sequentially based on ANOVA conducted on the fitted models. We tested for the significance of including the transects as random effects using linear mixed effect modelling (with the ‘nlme’ package, Pinheiro et al., 2017) to account for potential autocorrelation due to the spatial structure of the design. To do so, we fitted the full models with and without random effect and compared the fit using AIC_c_. Including random effects did not improve the fit, and so the backwards stepwise regressions were conducted on the simple linear models.

### 2.8 Statistical analyses of microbial community composition and structure

The Illumina sequenced data (GenBank BioProject Accession number: PRJNA762549) were processed through a bespoke metabarcoding bioinformatics pipeline developed for demultiplexing and analysing environmental microbial communities (Toju et al., 2018). Details are provided in the Methods S1.

The resulting sample × OTU (operational taxonomic unit) matrix was populated with sample metadata and OTU taxonomic classification and processed through statistical analyses of community composition using the R package ‘phyloseq’ (McMurdie & Holmes, 2013) as follows. Rarefied bacterial and fungal data (*n* = 100 and 50, respectively) were used to calculate OTU alpha diversity and richness metrics. To analyse the differences in community composition with treatments, we performed ordination analyses using the Bray–Curtis distance matrix and a non‐metric multidimensional scaling method across three dimensions (method = ‘NMDS’, trymax = 100, *k* = 3). We used PERMANOVA using the ‘adonis’ function of the vegan package (Oksanen et al., 2013) to determine the differences in microbial community composition due to biome, depth and thermal environment treatments. We tested the satisfaction of PERMANOVA assumptions using Levene's permutation test for homogeneity of multivariate dispersions (Anderson et al., 2006) and found that within‐group variation was non‐significant in each pairwise comparison among all communities (except fungi in kānuka, where small sample numbers led to significant skew in within‐group dispersion).

To assess variance in the taxonomic composition of bacterial and fungal communities across the different thermal environments (distance from geothermal depression, *D*_i_) in each biome, we converted OTU counts to relative abundances and summarized the shifts for dominant taxa (top 10 most prevalent genera in each biome). We scanned the literature for information on these taxa to identify thermophilic and thermotolerant taxa (see Section 3).

## 3 RESULTS

### 3.1 Treatments and environmental variables

The changes in environmental variables for the different treatments (depth, biome and distance from the heated depression), and the results from ANOVA, are presented in Table 1. There were no statistically significant differences in soil volumetric water content (*W*_s_) among the treatments. MET decreased, as anticipated, with increasing distance from the active heated depression at the site but also varied with biome and depth. MET was higher in the kānuka biome than in the grassland. The treatments resulted in a range of MET of more than 31°C, from 16.9 ± 0.8°C in grassland topsoils (0–50 mm) sampled at 30 m (furthest distance from the heated depression), to 48.3 ± 5.1°C in kānuka soils sampled at the 50–100 mm depth at 2 m (closest distance from the heated depression).

**TABLE 1:** Three‐way ANOVAs on environmental variables measured at the different distances from the geothermally active depression (distance, *D*_i_) in kānuka and grassland biomes (*B*) and at the two sampling depths (0–50 and 50–100 mm, *D*_e_). MET, mean environmental temperature, is the soil temperature at each sampling point for averaged over the 4 months prior to sampling; *W*_s_, soil volumetric water content, was measured just prior to sampling. Values are mean ± SEs (*n* = 3)

Biome (B)	Depth (*D*_e_)	Distance (*D*_i_)	MET (°C)	*W*_s_ (m^3^ m^−3^)	pH	C (%)	N (%)	C:N
Grassland	0–50 mm	2 m	24.2 ± 1.7	0.35 ± 0.09	4.1 ± 0.1	15.1 ± 4.8	0.89 ± 0.16	16.0 ± 2.5
		10 m	20.9 ± 1.5	0.31 ± 0.09	4.5 ± 0.1	8.3 ± 1.6	0.64 ± 0.12	13.0 ± 0.0
		30 m	16.9 ± 0.8	0.42 ± 0.08	4.8 ± 0.1	9.8 ± 2.5	0.78 ± 0.21	12.3 ± 0.3
	50–100 mm	2 m	31.6 ± 2.8	0.35 ± 0.07	4.5 ± 0.1	5.0 ± 1.3	0.37 ± 0.09	13.0 ± 0.6
		10 m	24.7 ± 2.4	0.30 ± 0.11	4.7 ± 0.1	3.8 ± 1.8	0.29 ± 0.12	12.7 ± 0.7
		30 m	19.6 ± 0.8	0.20 ± 0.02	4.9 ± 0.1	4.1 ± 1.3	0.29 ± 0.10	15.0 ± 1.0
Kānuka	0–50 mm	2 m	35.8 ± 0.3	0.28 ± 0.05	3.1 ± 0.1	6.3 ± 1.3	0.26 ± 0.04	23.7 ± 1.8
		10 m	30.8 ± 0.7	0.38 ± 0.07	3.1 ± 0.2	27.9 ± 8.7	0.98 ± 0.10	27.0 ± 5.5
		30 m	21.8 ± 0.2	0.40 ± 0.03	3.5 ± 0.4	32.3 ± 5.6	1.45 ± 0.12	22.7 ± 5.2
	50–100 mm	2 m	48.3 ± 5.1	0.29 ± 0.01	3.4 ± 0.1	1.2 ± 0.1	0.07 ± 0.01	18.5 ± 1.5
		10 m	38.8 ± 1.3	0.34 ± 0.05	3.1 ± 0.2	8.4 ± 4.0	0.33 ± 0.1	23.0 ± 3.5
		30 m	27.8 ± 0.5	0.21 ± 0.01	4.2 ± 0.4	6.6 ± 2.3	0.42 ± 0.13	14.7 ± 2.2
Significant terms			*B*, *D*_e_, *D*_i_	None	*B*, *D*_e_, *D*_i_	*B*, *D*_e_	*D*_e_, *D*_i_	*B*
Significant interactions			B*D_i_	None	None	B*D_e_, B*D_i_	B*D_i_	None
*p*‐value			<.0001	n.s.	<.0001	<.0001	<.0001	<.01
*R* ^2^			.89	.1	.83	.67	.72	.49

pH was also significantly affected by all three treatments, but the differences were strongest between biomes (Table 1) with a lower pH in the kānuka biomes. C concentrations changed significantly with sampling depth (with mean C of 16.6 ± 2.9% and 5.1 ± 0.9% for 0–50 and 50–100 mm depth, respectively) but also with biome (with mean C of 14.5 ± 3.4% and 7.7 ± 1.3% for kānuka and grassland, respectively). N concentrations also decreased with increasing depth (mean N of 0.83 ± 0.10% and 0.31 ± 0.04% for 0–50 and 50–100 mm depth, respectively) and were affected by the interaction between Biome and Distance from heated depression treatments. The C:N ratio changed significantly only with biome, being much lower in grassland (13.7 ± 0.5) than for kānuka (21.8 ± 1.6).

Mean environmental temperature was correlated with both pH and C:N ratio, but not with the other variables. As a result, overall, the combination of treatments resulted in a range of environments characterized by differences not only in environmental temperatures (MET) but also in pH and C:N ratio.

### 3.2 Treatments and microbial communities

**FIGURE 1:**
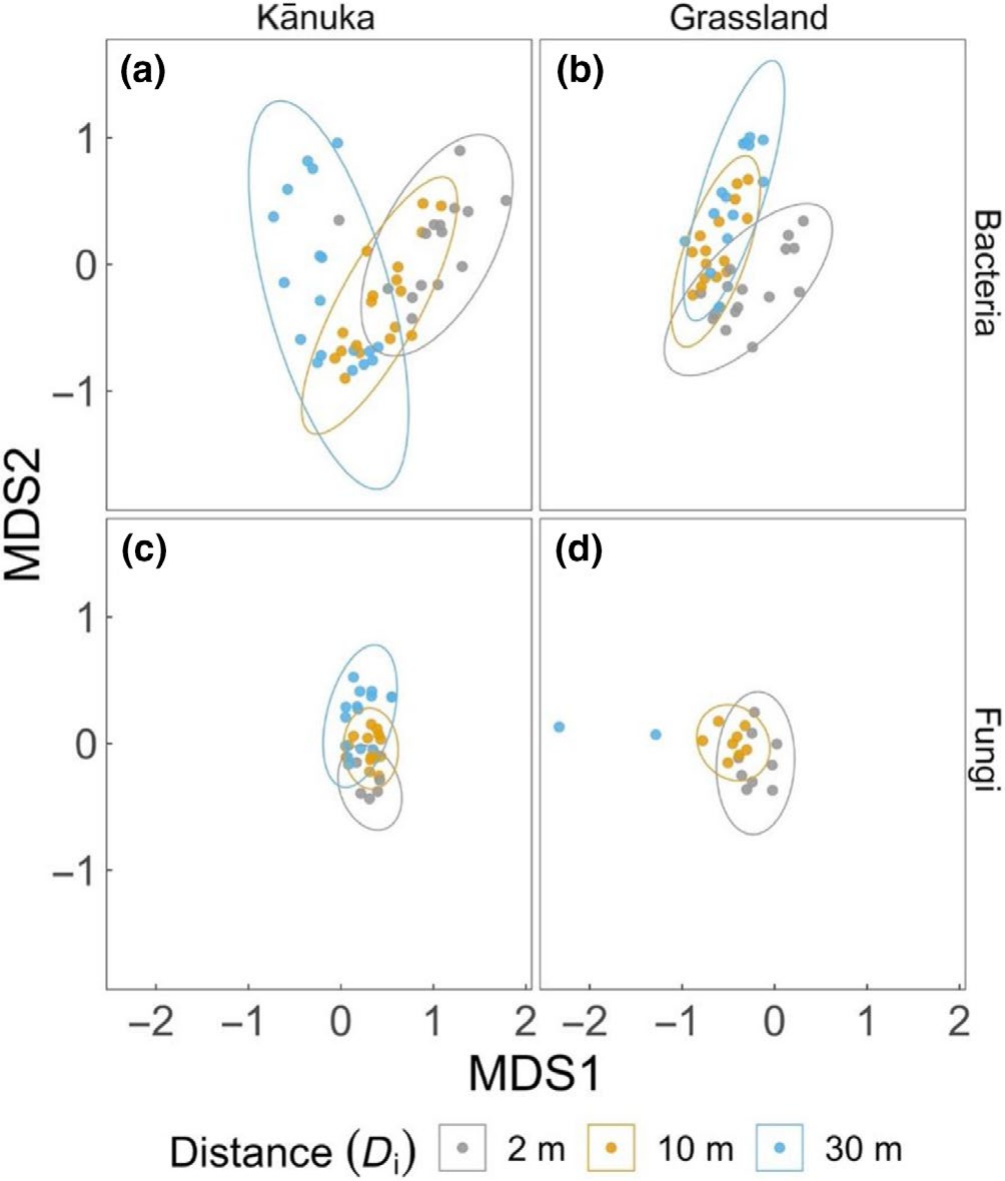
Microbial community composition shifts with temperature as a factor of distance from the geothermal‐heated depression. Non‐parametric multidimensional scaling (MDS) plots visualize the differences in bacterial (panels a and b) and fungal (panels c and d) community composition across the two biomes (grassland and kānuka) represented against the first two dimensions (MDS axes 1 and 2). The communities are strongly clustered within the 95% confidence ellipses across the distances (*D*_i_), with overlaps indicating compositional similarity and non‐overlaps indicating compositional dissimilarity. In each panel, the greatest dissimilarity is observed between the communities derived from the soils in the warmest (2 m distant from the heated depression) and coolest (30 m) locations

The bacterial and fungal community compositions were different for the kānuka and grassland biomes (Figure S2; Bacteria: *F* = 6.885, *R* ^2^ = .067, *p* = .001; Fungi: *F* = 7.1603, *R* ^2^ = .123, *p* = .001). Moreover, within each biome, the microbial community composition differed significantly across distances from the heated depression (Figure 1; Bacteria: *F* = 2.604, *R* ^2^ = .108, *p* = .001 for grassland and *F* = 5.569, *R* ^2^ = .188, *p* = .001 for kānuka; Fungi: *F* = 2.793, *R* ^2^ = .259, *p* = .001 for grassland and *F* = 4.522, *R* ^2^ = .226, *p* = .001 for kānuka).

The genera dominating the fungal and bacterial communities from grassland and kānuka biomes are presented in Figure S3. Grassland soil fungal communities were dominated by *Penicillium* sp., a versatile fungi regularly isolated from geothermally active sites (Redman et al., 1999). Kānuka soil fungal communities were dominated by *Pisolithus* sp., a geothermal specialist and ectomychorrhizal associate of prostate kānuka (Moyersoen & Beever, 2004). For bacteria, grassland soils were dominated by mesophilic bacteria such as *Conexibacter*, Gp1, Gp3, thermotolerant *Rhodoplanes*, thermophilic *Ktedonobacter*, lithotrophic WPS‐2 bacteria as well as ammonia‐oxidizing archaea *Nitrosphaera* (Hiraishi, 2017; Komaki et al., 2016; Sheremet et al., 2020; Tourna et al., 2011). In addition to some of the species dominating grassland soils, kānuka soils hosted acidophilic and psychrotolerant *Acidisoma* (Belova et al., 2009) and, methanogenic archaea *Methanomassilicoccus* (Kröninger et al., 2017), as well as three unique thermophiles: *Syntrophothermus*, *Thermogymnomonas* and *Thermosphaera*.

Shifts in the relative proportions of the taxa with the different distances from the heated depression (shown in Figure S4 for bacterial communities and Figure S5 for fungal communities) were observed, with some genera increasing and others decreasing, but overall showing an increasing relative abundance of thermophiles with decreasing distance (increasing MET) from the heated depression, particularly for kānuka. The relative abundances of the three kānuka bacterial thermophile species (*Syntrophothermus*, *Thermogymnomonas* and *Thermosphaera*) increased with decreasing distance from the heated depression (increasing MET). In grassland soils, *Penicillium* was dominant for the 2 and 30 m distances, but not the 10 m distance with intermediate MET, where psychrotolerant yeast *Solicoccozyma* (Stosiek et al., 2019), and moulds known for heat‐resistant spores (*Aspergillus* and *Talaromyces* sp.; Sørhaug, 2011) were found. In the fungal community of the kānuka biome, the dominant thermophilic *Pisolithus* sp. increased in relative abundance with decreasing distance from the heated depression. At the 30 m distance for the kānuka soils, *Penicillium* and other mesophilic species such as *Mortierella*, *Descole* and *Leptodontidium* were able to persist, but with their relative abundances lower than for the 2 m distance.

### 3.3 Treatments and fitted MMRT parameters

Macromolecular rate theory (Equation 1) provided a better fit to the data (lower AIC_c_) than the Lloyd & Taylor model (Equation 5) with differences in AIC_c_ all larger than 49. The changes in the MMRT parameters $\Delta H_{T_{0}}^{‡}$, $\Delta C_{P}^{‡}$, *T*_opt_, *T*_inf_ and *R*_25_ with the different treatments, as well as results from ANOVA, are presented in Table 2 and are described below. The changes in the Lloyd & Taylor parameters *R*_10_ and *E*_0_ are presented in Table S1.

**TABLE 2:** Three‐way ANOVAs on fitted macromolecular rate theory parameters measured at the different distances from the geothermal‐heated depression (distance, *D*_i_) in kānuka and grassland biomes (*B*) and at the two sampling depths (0–50 and 50–100 mm, *D*_e_). Values are mean ± SEs (*n* = 3)

Biome (*B*)	Depth (*D*_e_)	Distance (*D*_i_)	$\Delta H_{T_{0}}^{‡}$(kJ mol^−1^)	$\Delta C_{P}^{‡}$(kJ mol^−1^ K^−1^)	*T*_inf_ (°C)	*T*_opt_ (°C)	*R*_25_ (µmol CO_2_ g soil^−1^ min^−1^)
Grassland	0–50 mm	2 m	73.4 ± 3.2	−1.4 ± 0.2	54.2 ± 3.9	82.4 ± 6.6	23.9 ± 6.4
		10 m	65.8 ± 6.6	−2.6 ± 0.4	34.9 ± 4.6	54.0 ± 6.6	23.8 ± 7.2
		30 m	68.5 ± 2.3	−1.3 ± 0.1	53.6 ± 6.3	82.5 ± 8.7	40.8 ± 8.9
	50–100 mm	2 m	71.0 ± 4.1	−2.9 ± 0.3	33.7 ± 2.8	51.3 ± 3.8	12.8 ± 1.4
		10 m	60.0 ± 3.9	−3.2 ± 0.9	31.6 ± 5.4	49.8 ± 9.6	9.4 ± 3.1
		30 m	49.7 ± 9.8	−3.4 ± 1.7	32.2 ± 6.5	51.5 ± 10.9	8.3 ± 0.7
Kānuka	0–50 mm	2 m	65.8 ± 2.1	−1.9 ± 0.4	41.9 ± 6.4	65.4 ± 9.7	16.8 ± 4.7
		10 m	75.1 ± 1.7	−1.3 ± 0.2	57.8 ± 6.3	60.1 ± 8.8	35.1 ± 6.4
		30 m	77.7 ± 1.7	−1.1 ± 0.2	69.3 ± 8.1	103.1 ± 12.1	53.5 ± 9.1
	50–100 mm	2 m	38.6 ± 4.5	−1.3 ± 0.4	34.2 ± 10.0	62.6 ± 16.3	4.3 ± 0.8
		10 m	61.9 ± 2.4	−1.9 ± 0.2	37.7 ± 1.1	60.1 ± 2.1	11.9 ± 2.1
		30 m	59.2 ± 0.7	−2.1 ± 0.2	34.4 ± 1.8	55.4 ± 3.3	15.8 ± 2.1
Significant terms			*D*_e_	*D*_e_, *B*	*D*_e_	*D*_e_, *B*, *D*_i_	*D*_e_, *D*_i_
Significant interactions			B*D_i_	None	*D*_e_*D_i_	*D*_e_*B*D_i_	*D*_e_*D_i_
*p*‐value			<.001	<.01	<.001	<.01	<.0001
*R* ^2^			.61	.28	.60	.73	.76

$\Delta C_{P}^{‡}$ varied significantly with biome (mean ± SE, *n* = 18, −2.5 ± 0.4 and −1.6 ± 0.1 kJ mol^−1^ K^−1^ for grassland and kānuka, respectively) and depth (*n* = 18, −1.6 ± 0.2 and −2.5 ± 0.4 kJ mol^−1^ K^−1^ for 0–50 and 50–100 mm depths, respectively) but not with distance from the heated depression, and with no significant interactions between treatments (Table 2). For all other parameters, significant interactions were found between different combinations of biome, distance from the heated depression and depth treatments (Table 2). Despite a clear increase in MET with decreasing distance from the heated depression along each transect (Table 1), distance from the heated depression did not influence any of these variables (except *R*_25_) in a consistent manner, with mean values being sometimes higher, lower or intermediate for the warmer treatment depending on which combination of biome and depth treatments is considered. *R*_25_ increased from 2 to 10 to 30 m from the heated for all cases except for the grassland biome at the 50–100 mm depth (Table 2). Overall, estimates for *T*_opt_ ranged above the measurement temperatures, with a mean ± SE (*n* = 3) minimum of 49.8 ± 9.6°C in the grassland biome at 10 m from the heated depression and 50–100 mm depth and a maximum of 103.1 ± 12.1°C in the kānuka biome at 30 m and 0–50 mm depth (Table 2). The depth treatment affected all parameters (Table 2).

**FIGURE 2:**
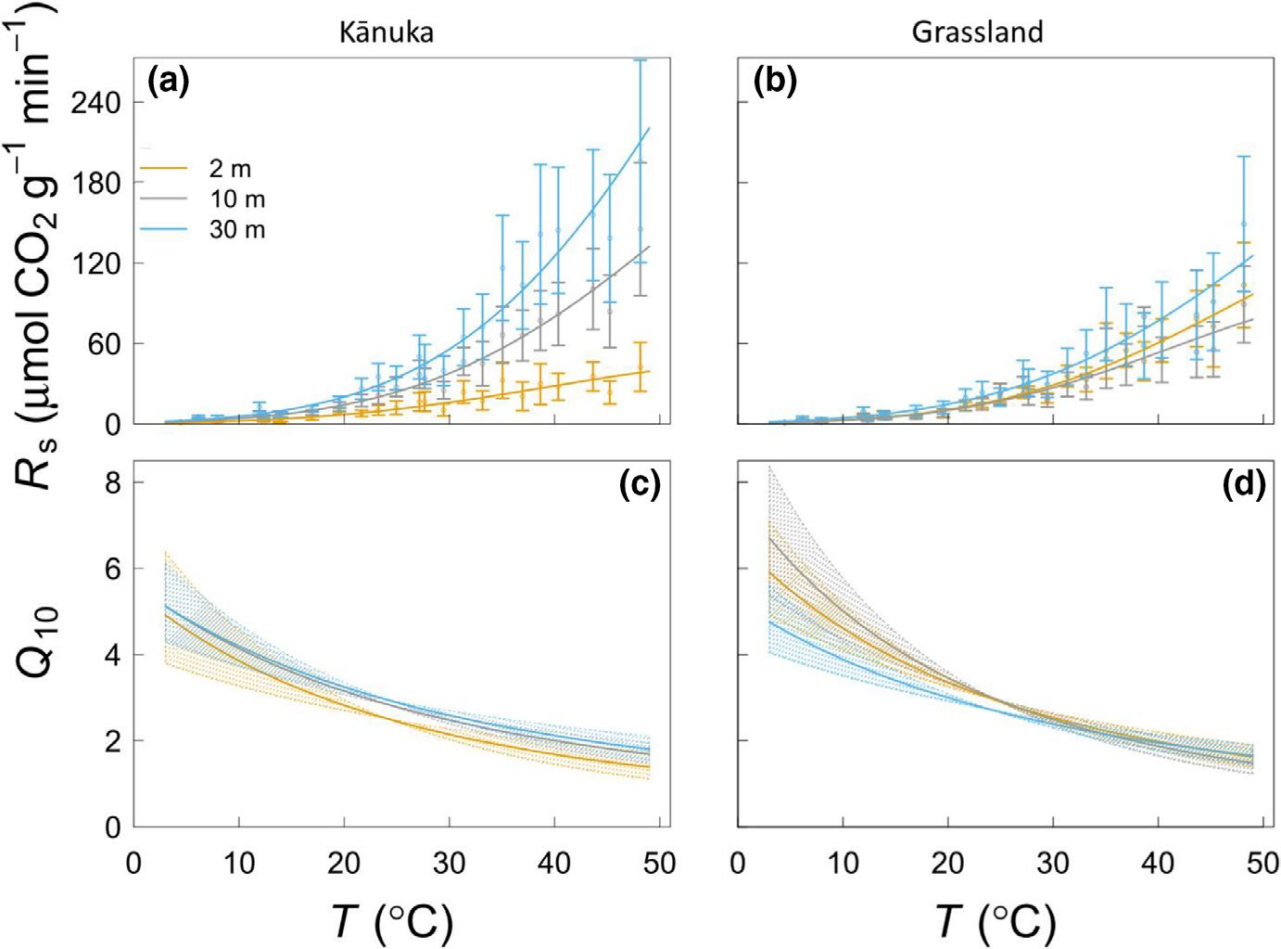
Temperature responses of microbial decomposition of SOM (*R*_s_; panels a and b) and relative temperature sensitivity (*Q*_10_; panels c and d) to temperature (*T*) for the different thermal environments obtained at 2, 10 and 30 m from the heated depression in kānuka (panels a and c) and grassland (panels b and d) biomes. In the panels (a) and (b), the points are mean values of *R*_s_ with both depth treatments confounded and the whiskers represent the standard error (*n* = 6). The lines in the panels (a) and (b) represent the fit of the macromolecular rate theory (MMRT) model (Equation 1). The lines in the panels (c) and (d) were calculated with Equation (6) using the estimated MMRT parameters. The shaded areas represent the error associated with *Q*_10_ calculations obtained from the standard errors (*n* = 6) of parameter estimates

As a result of the parameter variations, the temperature responses of *R*_s_ at the different distances from the heated depression appeared to be different for grassland and particularly kānuka (which also showed greater variation in MET; Figure 2a,b). This was the case mainly for the magnitude (regarding the absolute values of *R*_s_ at the measurement temperatures, informed by *R*_25_ and $\Delta H_{T_{0}}^{‡}$), but not for the relative temperature sensitivity (the relative change in *R*_s_ per unit increase in measurement temperature, informed by $\Delta C_{P}^{‡}$ and *Q*_10_) nor for the temperature optimum (*T*_opt_) and temperature at the highest sensitivity (*T*_inf_). Similarly to the *Q*_10_ calculated from MMRT (Figure 2c,d), the *Q*_10_ derived from the Lloyd & Taylor model (Equation 7) showed little differences in relation to the distances from the heated depression or between biomes, except for the kānuka biome at 2 m which appeared lower than for the other treatments (Figure S6).

### 3.4 Influence of environmental variables on MMRT parameters

Differences in all the MMRT parameters could be attributed to differences in C concentration and this alone explained 28%, 10%, 46%, 53% and 44% of the variability in $\Delta H_{T_{0}}^{‡}$, $\Delta C_{P}^{‡}$, *T*_opt_, *T*_inf_ and *R*_25_, respectively.

**FIGURE 3:**
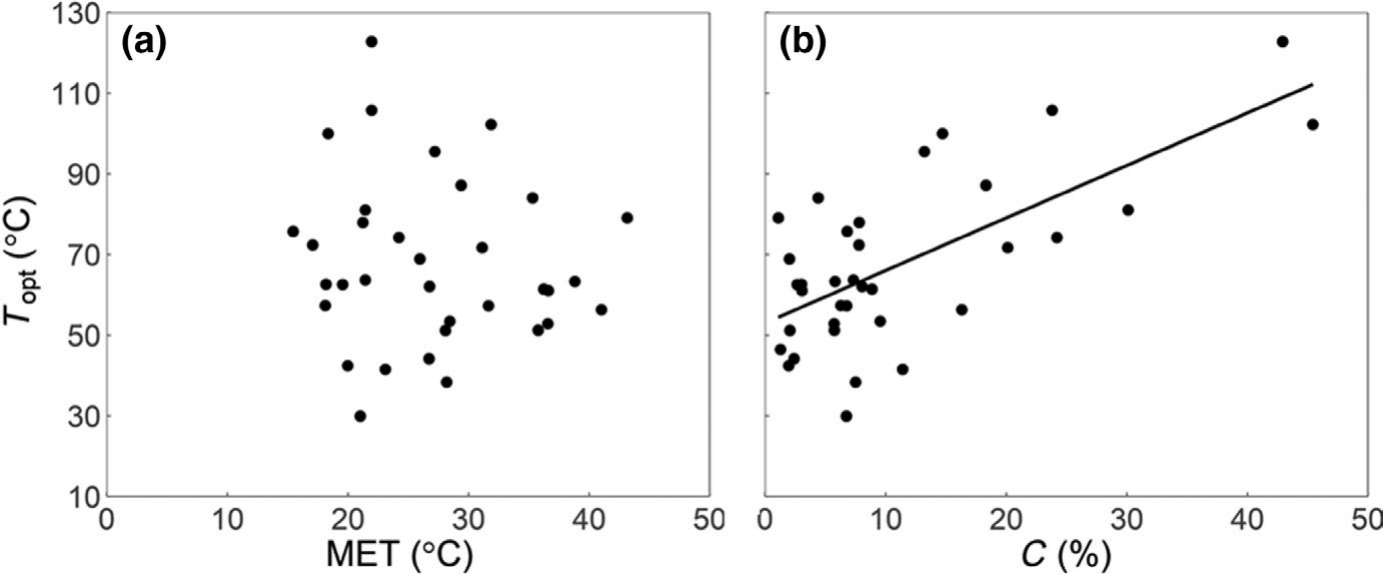
*T*_opt_ (optimum temperature calculated from macromolecular rate theory fit) as a function of mean environmental temperature (MET, a) and soil carbon (C) concentration (b). The line indicates a significant linear regression fit between C concentration and *T*_opt_

$\Delta H_{T_{0}}^{‡}$ increased significantly with C concentration (*F*_1,31_ = 12.1, *p* < .01) and decreased with MET (*F*_1,31_ = 6.0, *p* = .02). Only MET and C concentration influenced $\Delta H_{T_{0}}^{‡}$ significantly, with no interaction, and explained 35% of the variability. *T*_opt_ was affected significantly only by C concentration (Figure 3a), showing a positive effect with a slope estimate of 1.3 ± 0.2°C %C^−1^ (*F*_1,31_ = 26.4, *p* < .0001) with no effect of MET (Figure 3b; with a slope estimate of −0.4 ± 0.4°C °C^−1^, *F*_1,31_ = 1.8, *p* = .2). This was also the case for $\Delta C_{P}^{‡}$ and *T*_inf_, which both increased with C concentration (*F*_1,31_ = 4.6, *p* = .04 for $\Delta C_{P}^{‡}$ and *F*_1,31_ = 34.4, *p* < .0001 for *T*_inf_) and did not vary with MET (*F*_1,31_ = 0.4, *p* = .5 for $\Delta C_{P}^{‡}$ and *F*_1,31_ = 3.9, *p* = .06 for *T*_inf_). Similar to $\Delta C_{P}^{‡}$, *E*_0_, the parameter in the Lloyd & Taylor model related to relative temperature sensitivity, increased with C concentration (*F*_1,31_ = 19.5, *p* < .0001) but was not significantly affected by MET (*F*_1,31_ = 3.4, *p* = .07).

**FIGURE 4:**
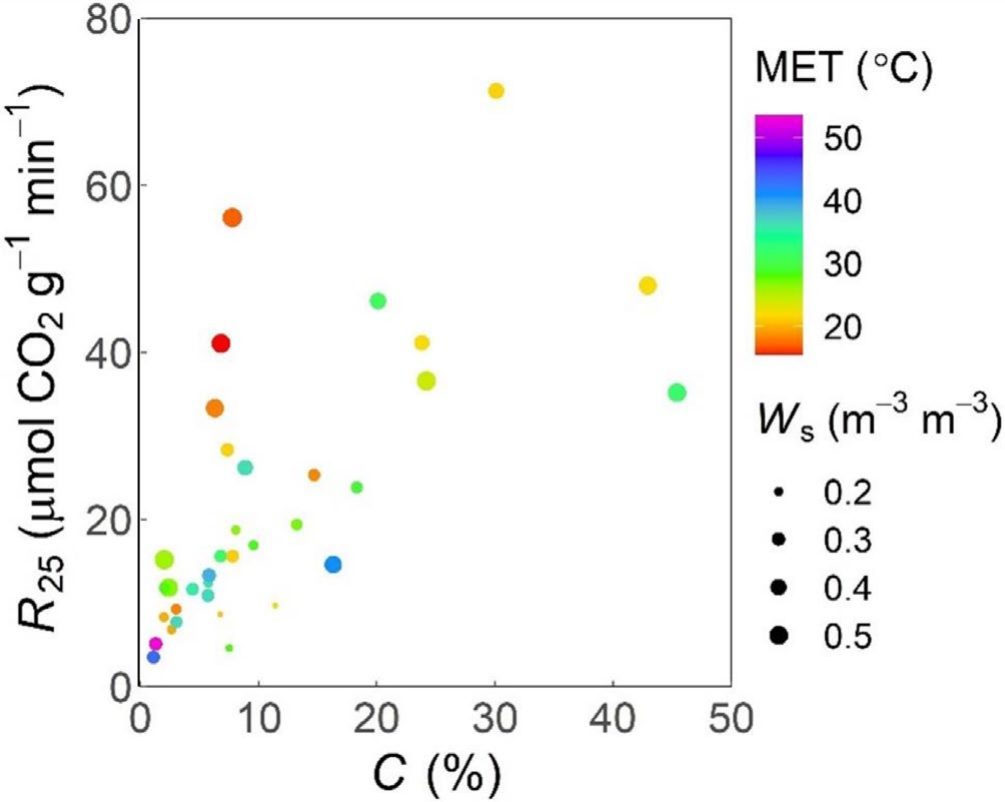
*R*_25_ (respiration rate at 25°C calculated from macromolecular rate theory, Equation 4) in relation to soil carbon (C) concentration. The colours represent the mean environmental temperatures (MET) and the sizes indicate the soil volumetric water content (*W*_s_) at the time of the measurements

*R*_25_ was significantly affected by a combination of C concentration (which explained most variation with a strong positive effect), *W*_s_ (positive effect) and MET (positive effect, despite lower mean values at 2 m than at 30 m from the heated depression) (Figure 4). The triple interaction was not significant, but two‐way interactions were significant between C concentration and MET (*F*_1,31_ = 6.1, *p* = .02), C concentration and *W*_s_ (*F*_1,31_ = 5.3, *p* = .03) and MET and *W*_s_ (*F*_1,31_ = 5.0, *p* < .03). *R*_25_ was the only parameter significantly affected by *W*_s_.

## 4 DISCUSSION

We characterized the temperature response of microbial decomposition of SOM (using MMRT) and the composition of microbial communities (using metabarcoding) from soils sampled at increasing distances from a geothermally heated depression in two biomes and at two sampling depths. The sampling design resulted in a range of thermal environments varying widely in soil temperature and soil C concentration, but also in pH, N concentrations and soil C:N ratios. C concentration and MET varied independently across the treatments so that it was possible to assess the interacting effects of those two variables on the temperature response parameters. In support of our first hypothesis, we found that the different thermal environments were hosting microbial communities with distinct compositions, with thermophilic and thermotolerant genera decreasing in relative abundance with increasing distances from the heated depression (and increasing MET). However, MET had no detectable influence on the MMRT parameters influencing the relative temperature sensitivity ($\Delta C_{P}^{‡}$ and $\Delta H_{T_{0}}^{‡}$, Equation 6), leading to similar *Q*_10_ values along the transects of decreasing MET. Similarly, *T*_opt_ was not influenced by MET. We must therefore reject our second hypothesis that the microbial communities would have adapted to their environmental temperature leading to a lower relative temperature sensitivity of SOM decomposition. Interestingly, C concentration was positively correlated with the rate of respiration at a standard temperature of 25°C (*R*_25_) and $\Delta H_{T_{0}}^{‡}$, determining the magnitude of the change in *R*_s_. This occurred concurrently, with C concentration being positively correlated with *T*_opt_ and $\Delta C_{P}^{‡}$, albeit only marginally for the latter (10% of the variance explained, against 46% for *T*_opt_).

### 4.1 Experimental conditions at the geothermally active site

Geothermally heated ecosystems have been recognized recently as naturally occurring warming experiments that allow interpretation of the long‐term adaptation of undisturbed biomes to temperature gradients (O'Gorman et al., 2014). Our 31°C temperature range in MET is comparable with gradients of 20 or 30°C along distances of tens of metres at other geothermally heated sites (O’Gorman et al., 2014; Sigurdsson et al., 2016). While we did not measure soil temperature throughout the year, our 4 month average characterization of soil temperatures is comparable to mean annual temperatures ranging from 16.7 to 50.4°C reported earlier at the same site (Nishar et al., 2017). pH was lower at our site compared to that at other geothermal sites used for climate warming experiments (O'Gorman et al., 2014; Sigurdsson et al., 2016) but the negative relationship between pH and MET was observed previously (Nishar et al., 2017; O'Gorman et al., 2014). C concentration varied widely between measurement locations and was particularly high at some locations in the kānuka biome (>30%). This was probably due to the incorporation of nearly undecomposed leaf litter from recent leaf fall into the topsoil.

### 4.2 Thermal adaptation of microbial community composition

The species composition of microbial communities changed with distance from the heated geothermal depression for both the grassland and kānuka biomes. This contrasts with previous studies where no differences were found in microbial community composition sampled from sites with a 9°C warming along geothermal spatial gradients in Iceland (Radujković et al., 2018; Walker et al., 2018). Our findings are consistent with observations over large latitudinal gradients showing temperature‐driven changes in microbial community composition (Deslippe et al., 2012; Nottingham et al., 2018; Zhou et al., 2016). Despite being spatially co‐located (<50 m distant), the microbial communities within our two biomes were distinct, with the differences probably driven by the differences in vegetation characteristics and C inputs. Specifically, the kānuka biome hosted geothermal specialist ectomycorrhizal fungi (*Pisolithus*), which were rare in grassland soils, suggesting a strong symbiotic relationship with prostate kānuka. Fungal spores disperse readily, but in the absence of its symbiotic partner, mycorrhizal fungi cannot often persist (Moyersoen et al., 2003).

The kānuka biome also experienced a greater range in MET across the sampled locations. This further influenced the structure and functional profile of both the bacterial and fungal communities. Not only were more thermophilic and thermotolerant bacteria and fungi dominant in the kānuka biome, but they also decreased in relative abundance with environmental temperature (increasing distance from the heated depression). This suggests specialization of species composition to the thermal environment (Jacob et al., 2018). Microbial dispersal and evolutionary dynamics explain this as recruitment of thermally adapted species to geothermally active local sites or the emergence of hotspots for local thermal‐adaptation, despite the prevalence of mesophilic species in the regional pool (Norris et al., 2002). While these trends are expected (Deslippe et al., 2012; Rinnan et al., 2007), they represent long‐term adaptive processes that may partially apply to mesophilic soil communities experiencing short‐ or medium‐term warming (Radujković et al., 2018; Romero‐Olivares et al., 2017). However, over the longer term, our findings indicate that, with increasing temperatures associated with climate warming, thermally adapted communities may feature more significantly in future soil communities.

### 4.3 MMRT parameters as microbial thermal traits

The parameter *T*_opt_ defines the temperature at which *R*_s_ reaches its maximum. The value of *T*_opt_ can reach well above 50°C, beyond biologically relevant temperatures for most natural ecosystems (Robinson et al., 2017; 2020; Schipper et al., 2019). However, *T*_opt_ constrains the curvature of the temperature response (positive correlation with $\Delta C_{P}^{‡}$) and has therefore direct relevance for microbial thermal adaptation (Alster et al., 2020). *T*_inf_, the temperature at which the relative (and absolute) temperature sensitivity is highest, also influences the curvature of the temperature response and has been hypothesized to be under strong selective pressure with shifting temperatures (Prentice et al., 2020), and is therefore also relevant in the context of microbial thermal adaptation. Our estimates of *T*_opt_ ranged from 49.8 to 103°C, the high end being well above the range observed previously for soil respiration (Robinson et al., 2017; Schipper et al., 2014, 2019). *T*_inf_ was strongly correlated with *T*_opt_ and was similarly influenced by environmental variations, therefore pointing to the same overall interpretation. Our estimates of $\Delta C_{P}^{‡}$ (ranging from −3.4 to −1.1 J mol^−1^ K^−1^) were similar to those from previous studies, with a range of −3.1 to −1.6 J mol^−1^ K^−1^ for two comparable studies (Robinson et al., 2017; Schipper et al., 2019). It has been shown that fitting MMRT to data where the range of measurement temperatures does not reach *T*_opt_ may lead to overestimation of *T*_opt_ and $\Delta C_{P}^{‡}$ (Alster et al., 2018). This was the case of the majority for our 35 response curves and may explain the high estimates of *T*_opt_ and $\Delta C_{P}^{‡}$ observed for some of the curves. For this reason, as a comparison, we conducted an analysis of the parameter from the Lloyd & Taylor model most related to the temperature sensitivity, *E*_0_, and compared *Q*_10_ values estimated from the MMRT and Lloyd & Taylor models. Results from this analysis led to identical conclusions as those made from MMRT observations. Therefore, although absolute values of MMRT parameters may have been overestimated, we argue that our analysis provided reliable estimates for comparisons between our treatments and across the environmental variation at our site.

### 4.4 MMRT, warming, substrate quality and quantity

Alster et al. (2020) proposed that $\Delta C_{P}^{‡}$ and *T*_opt_ are the temperature response traits most relevant to test hypotheses related to the thermal adaptation of microbial communities. We hypothesized that *T*_opt_ and $\Delta C_{P}^{‡}$ would both increase with warming due to a thermal selective pressure leading to increased abundance of microbes producing warm‐adapted (rigid) enzymes to constrain reaction rates when temperature increases (Hochachka & Somero, 2002), in line with an attenuating effect of thermal adaptation on soil C losses (Bradford et al., 2019). However, *T*_opt_ and $\Delta C_{P}^{‡}$ did not vary with MET, so we must reject this hypothesis.

The variables with the most influence on the parameters derived from MMRT was C concentration. However, soil C:N ratios and N concentrations were positively correlated with C concentration, and so we are unable to distinguish between the influence of these variables on the MMRT parameters. Nonetheless, our findings can explain the positive influence of C concentration on the respiration rate at 25°C (*R*_25_) and $\Delta H_{T_{0}}^{‡}$, a parameter related to the magnitude of change in respiration rates. Substrate deprivation leads to decreased metabolism and respiration in heterotrophic microbes (Bradford, 2013). As a result, the response of microbial respiration to temperature is dependent on substrate availability, with an increase in the response as C concentration increase and substrates become more abundant (Davidson & Janssens, 2006). Furthermore, higher substrate availability and respiration rate are positively correlated with microbial biomass (Allison et al., 2010; Bradford, 2013), so, an increase in microbial biomass with higher C concentration and substrate availability in the treatments at our site would also explain increases in *R*_25_ and $\Delta H_{T_{0}}^{‡}$.

*T*_opt_ and $\Delta C_{P}^{‡}$ were also influenced by C concentration. Again, this effect could be attributed to the correlation with C:N ratios (or N concentrations). Both a positive influence of C:N ratios and C concentration on *T*_opt_ and $\Delta C_{P}^{‡}$ can be supported theoretically. Indeed, when substrates are accessible by microbes, there is a positive relationship between the recalcitrance of substrates (of which C:N is often taken as an indicator) and the temperature sensitivity of their decomposition (Conant et al., 2011; Davidson & Janssens, 2006; Fierer et al., 2005). Decreases in the substrate quality due to the depletion of labile substrates have been proposed as an explanation for compositional shifts in microbial communities and increases in temperature sensitivities that occur as an indirect consequence of warming (Bai et al., 2017; Karhu et al., 2014; Pold et al., 2015). Moreover, the temperature response of microbial decomposition is constrained by both biological enzymatic reaction and chemical reactions regulating substrate exchange between the solid and aqueous phases of the soil (Conant et al., 2011). Numa et al. (2021) and Schipper et al. (2019) argued that the resulting temperature response would be a combination of an Arrhenius‐driven temperature response of sorption/desorption and diffusion and an MMRT‐driven response of the biological process. If C concentration at our site were positively related to physicochemical protection of C substrate (Kirschbaum et al., 2020), an increasing contribution of Arrhenius‐driven reactions could have resulted, and therefore a lower observed $\Delta C_{P}^{‡}$ (Schipper et al., 2019). Having disentangled the effects of warming from those of substrate quality and quantity (MET varied independently from C concentration and C:N ratios), our data suggest that shifts in substrate quality and/or quantity may exert a selective pressure greater than that for temperature in the composition of communities with distinct temperature response parameters and relative temperature sensitivities.

### 4.5 Thermal adaptation of microbial decomposition function

We found no evidence for thermal adaptation of microbial decomposition of SOM, in contrast to other recent studies where respiration was observed to be downregulated over long‐term warming in conditions of unlimited substrate availability (Bradford et al., 2019; Dacal et al., 2019); this occurred despite temperature‐driven shifts in the composition of microbial communities. However, changes in the temperature response of soil respiration observed in our study were largely driven by shifts in substrate quality and/or quantity. Rates and the magnitude of changes in soil respiration increased with substrate concentration, possibly partly as a consequence of increasing microbial biomass (which we did not measure), consistent with previous observations in soils along geothermal gradients (Walker et al., 2018). Surprisingly, the variations in *T*_opt_ and $\Delta C_{P}^{‡}$ were also largely driven by substrate availability, with no detectable influence of environmental warming.

Our novel approach used a unique combination of measurements at a natural geothermally heated site with independent variability in soil temperatures and substrate quality and quantity. We derived MMRT parameters to determine intrinsic properties of microbial physiology at the community scale and characterized microbial community composition. As such, our study has provided new insights to understand long‐term thermal adaptation of microbial communities. Our findings suggest that, while long‐term warming selects for warm‐adapted taxa, substrate quality and quantity exert a stronger influence than temperature itself in selecting for distinct thermal response traits. This implies that observations of thermal adaptation in conditions of unlimited substrate supply may be unrealistic. The results have major implications for our understanding of soil microbial processes and the long‐term effects of climate warming on soil C dynamics and its feedback to climate change.

## CONFLICT OF INTEREST

The authors declare no conflict of interest.

## Supporting information

Supplementary Material

Supplementary Material

## Data Availability

The data that support the findings of this study are available from the corresponding author upon reasonable request. Metabarcoding data is available on GenBank Project Accession PRJNA762549: https://dataview.ncbi.nlm.nih.gov/object/PRJNA762549
