# Supplementary material for: Soil microbial sensitivity to temperature remains unchanged despite community compositional shifts along geothermal gradients
Source: Glob Chang Biol. 2021 Sep 28;27(23):6217–31. doi: 10.1111/gcb.15878 (PMC9293425; doi:10.1111/gcb.15878)
Supplement: Supplementary file 1 — Supplementary Material [file GCB-27-6217-s002.docx]

**Supplementary Figure S1.** Conceptual representation of the full factorial design of the study. Soils were sampled at 0-50 mm and 50-100 mm depth along transects with increasing distances from a geothermal active zone at 2, 10 and 30 m in areas covered with grassland and areas covered in kānuka stands. Mean seasonal temperature (MST) (mean soil temperature of the four months prior to the study, used here a proxy for mean annual temperature, MAT), decreased with increasing distance from the heated zone.


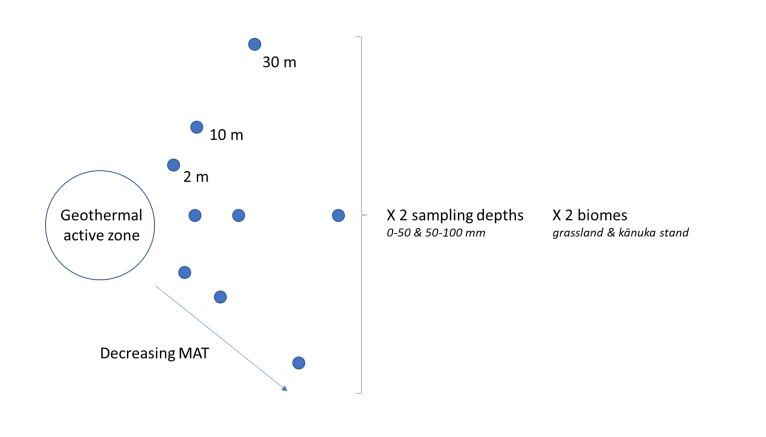


**Supplementary Figure S2:** **Distinct microbial communities observed across co-located but different biomes.** Non-parametric multidimensional scaling (nMDS) plots representing the compositional differences observed in the bacterial (left) and fungal (right) communities across the first two dimensions (MDS Axis 1 & 2).


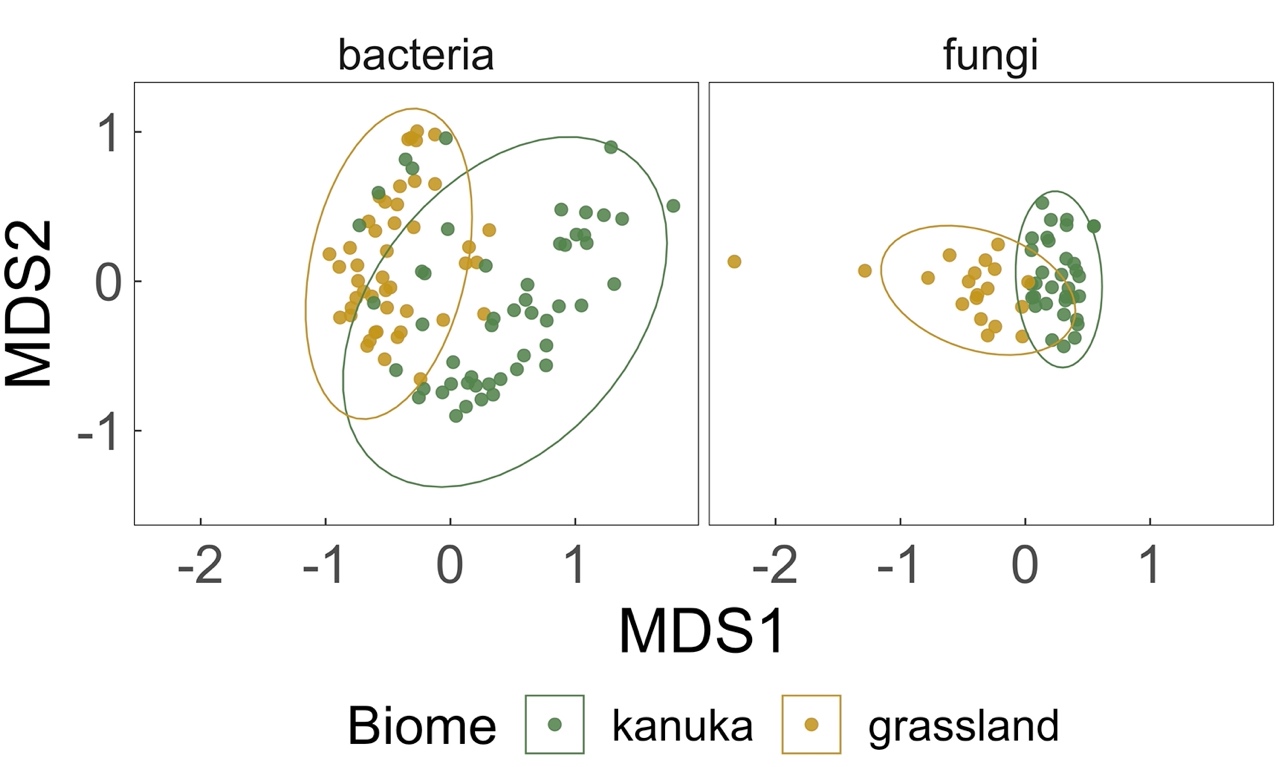


**Supplementary Figure S3: Abundance of microbial taxa in soil communities derived from the grassland and kanuka biomes.** Relative abundances of top 10 genera of bacteria (left) and fungi (right) vary between the two co-located soil biomes. This difference is especially important in fungal communities, where kanuka associated *Pisolithus* sp. replaces *Penicillium* and dominates the kanuka biome fungal community.

**
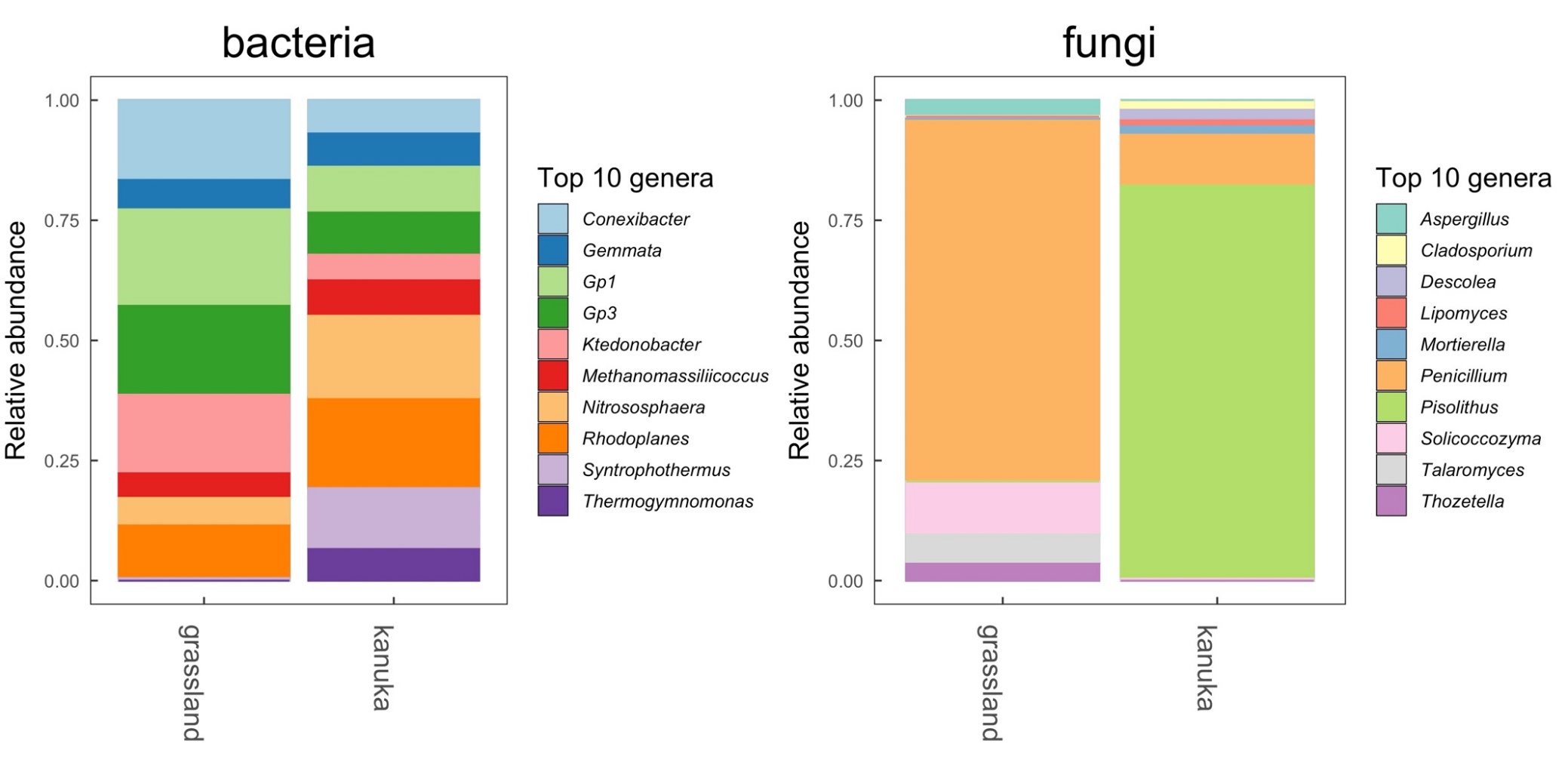
**

**Supplementary Figure S4:** S**hift in** d**ominant bacterial taxa with distance from the geothermally heated depression.** Relative abundance of top 10 genera across kanuka and grassland biomes change with temperature as designated as by different distances (Di). More notable changes are observed with the kanuka biome where a greater shift in MST was observed.


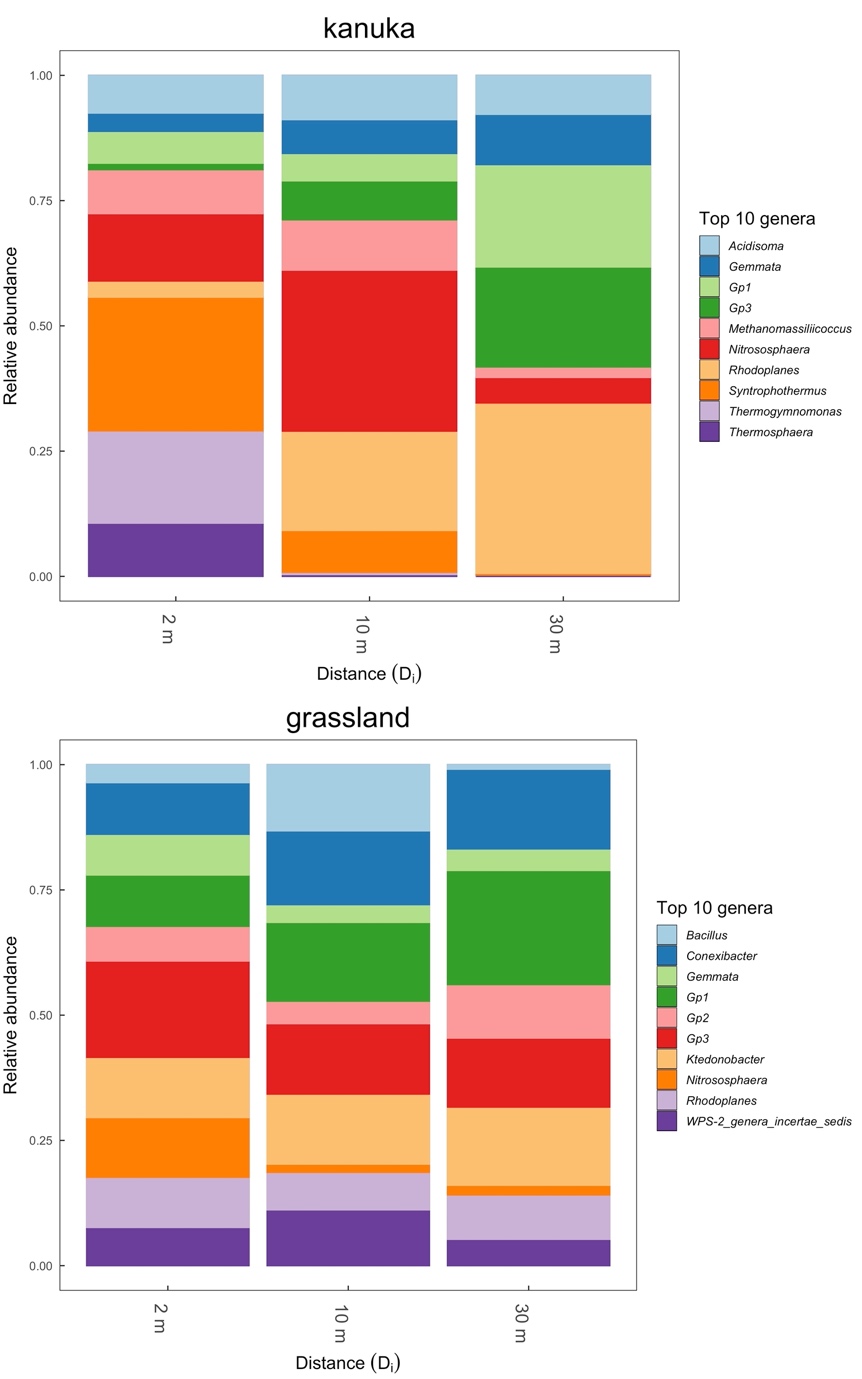


**Supplementary Figure S5:** **Shift in** d**ominant fungal taxa with distance from the geothermally heated depression.** Relative abundance of top 10 genera across kanuka and grassland biomes change with temperature as designated as by different distances (*D*_i_).


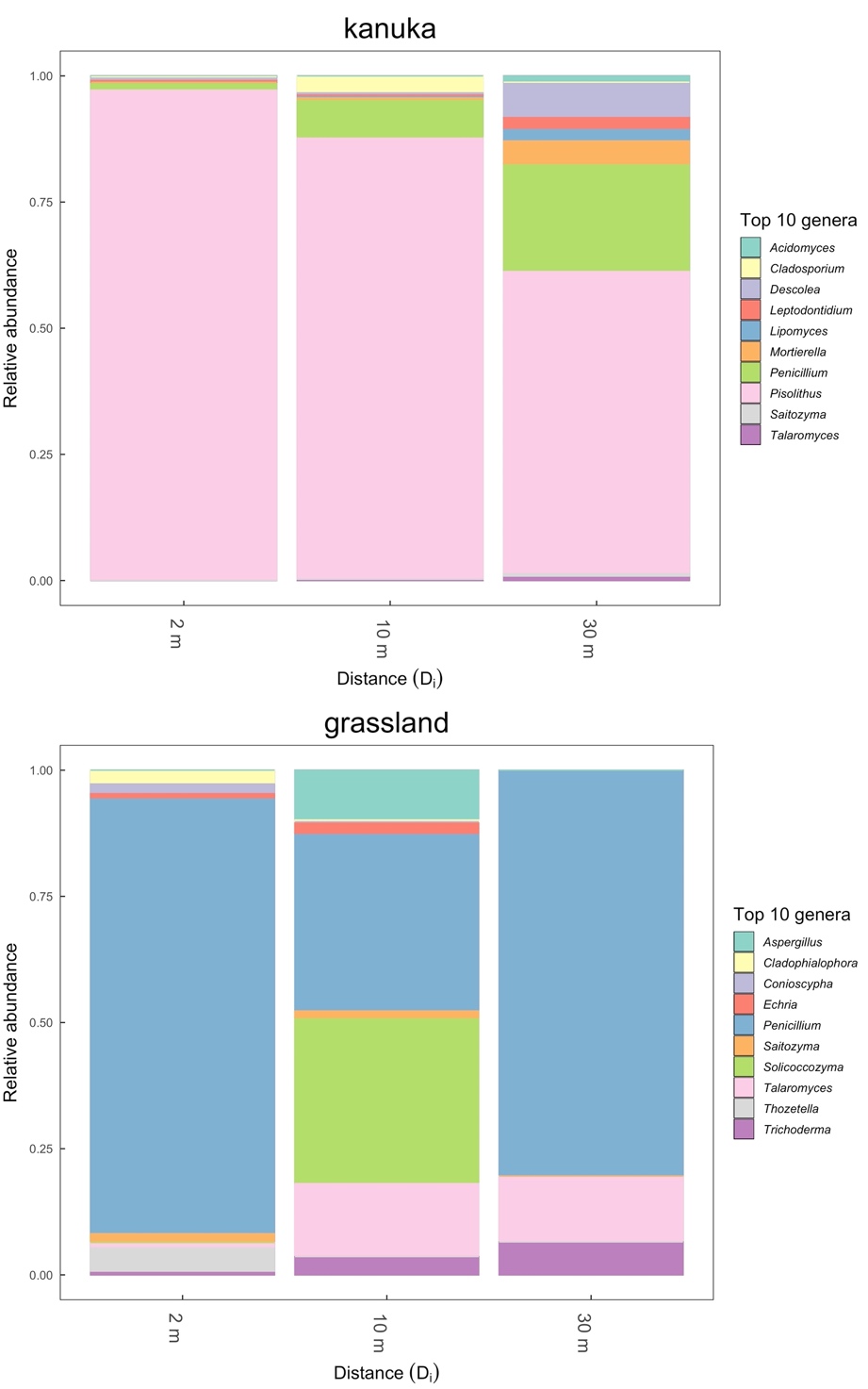


**Supplementary Figure S6:** Temperature responses of microbial decomposition of SOM (*R*_s_) (upper panels) and relative temperature sensitivity (*Q*_10_) (lower panels) to temperature (*T*) for the different thermal environments obtained at 2, 10 and 30 m from the heated depression in kānuka (left panels) and grassland (right panels) biomes. In the upper panels, the points are mean values of *R*_s_ with both depth treatments confounded and the whiskers represent the standard error (n = 6). The lines in the upper panels represent the fit of the Lloyd & Taylor model (Equation 5). The lines in the lower panels were calculated with Equation 7 using the estimated Lloyd & Taylor parameters. The shaded area represent the error associated with *Q*_10_ calculations obtained from the standard errors (n = 6) of parameter estimates. The lines for the *Q*_10_ at 2 and 30 m from the heated depression in the grassland biome nearly completely overlap.


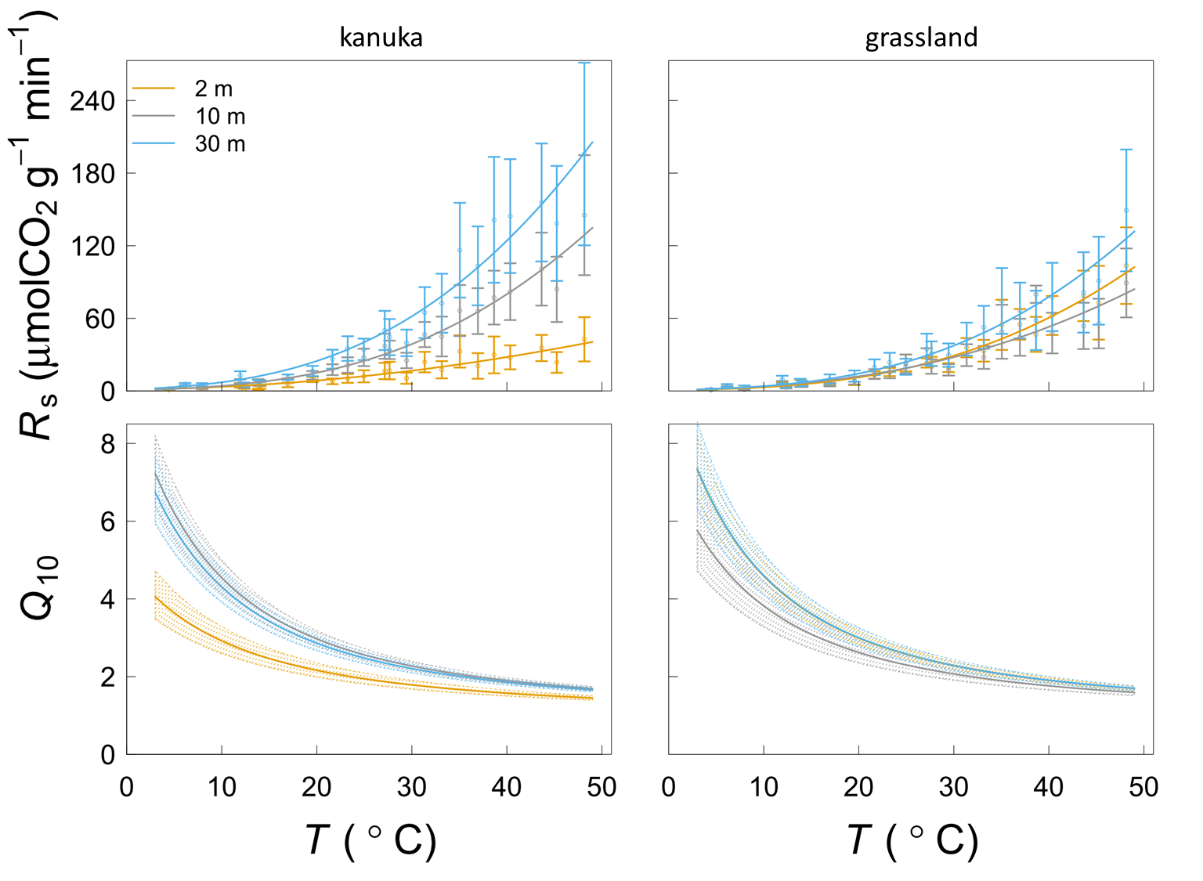


**Supplementary Table S1**. Three-way ANOVAs on fitted Lloyd & Taylor parameters measured at the different distances from the geothermal heated depression (Distance, *D*_i_) in kānuka and grassland biomes (B) and at the two sampling depths (0-50 and 50-100 mm, D_e_). Values are mean ± standard errors (n = 3).

| Biome (B) | Depth (*D*_e_) | Distance (*D*_i_) | *R*_10_ (µmolCO_2_ gsoil^-1^ min^-1^) | *E*_0_ (K) |
| --- | --- | --- | --- | --- |
| Grassland | 0–50 mm | 2 m | 3.6 ± 0.5 | 498 ±25 |
|  |  | 10 m | 5.7 ± 1.2 | 362 ± 59 |
|  |  | 30 m | 4.5 ± 2.6 | 480 ± 38 |
|  | 50–100 mm | 2 m | 2.9 ± 0.8 | 402 ± 64 |
|  |  | 10 m | 2.7 ± 0.6 | 306 ± 46 |
|  |  | 30 m | 2.9 ± 0.4 | 282 ± 59 |
| Kānuka | 0–50 mm | 2 m | 4.9 ± 2.1 | 388 ± 66 |
|  |  | 10 m | 5.5 ± 1.5 | 514 ± 43 |
|  |  | 30 m | 11.5 ± 2.6 | 474 ±33 |
|  | 50–100 mm | 2 m | 1.6 ± 0.5 | 255 ± 35 |
|  |  | 10 m | 3.5 ± 0.7 | 352 ± 22 |
|  |  | 30 m | 4.9 ± 0.5 | 328 ± 8 |
| Significant terms | | | D_e_, D_i_ | D_e_ |
| Significant interactions | | | none | B*D_i_ |
| *p*-value | | | < 0.001 | < 0.001 |
| R^2^ | | | 0.46 | 0.59 |
