# Supplementary material for: Soil microbial sensitivity to temperature remains unchanged despite community compositional shifts along geothermal gradients
Source: Glob Chang Biol. 2021 Sep 28;27(23):6217–31. doi: 10.1111/gcb.15878 (PMC9293425; doi:10.1111/gcb.15878)
Supplement: Supplementary file 2 — Supplementary Material [file GCB-27-6217-s001.docx]

**SUPPLEMTARY METHODS**

The Illumina sequencing data (GenBank BioProject Submission number: SUB9552103) were processed through a bespoke bioinformatics pipeline (Toju et al., 2018). Briefly, sequence read fastqs were generated from raw binary base call files using the Bcl2fastq2 tool (Illumina version 2.20), and demultiplexed using Claident pipeline (version 2018.05.08) (Tanabe & Toju, 2016). Paired forward and reverse reads were merged using pear (min overlap = 15 bp) and filtered for length (>300 bp) and quality (Q30) (Zhang et al., 2014). Clustering by sequence similarity (min identity = 0.97) to call operational taxonomic units (OTUs) was performed using Vsearch (2.4.3, Rognes et al., 2016), followed by stringent chimera removal using *de-novo* and reference-based approaches. Taxonomic assignment was performed using the custom reference databases and query-centric auto-*k-*nearest neighbour (QCauto) method built into Claident (Tanabe & Toju 2013) and summarised OTU diversity across samples to create OTU counts × sample matrix followed by filtering of low prevalence (<10 sequences), no taxonomic hits and low confidence OTUs.

The final sequencing dataset was comprised of a total of 97 soil samples that yielded 978 unique bacterial OTUs represented across 47 268 sequences and 65 soil samples that yielded 79 unique fungal OTUs represented across 12 558 sequences. Negative and extraction controls did not amplify and 3 out of 12 Synmock species did not amplify, with the remaining 9 returned sequences that were accurately attributed to mock community controls.

**REFERENCES**

Rognes, T., Flouri, T., Nichols, B., Quince, C., & Mahé, F. (2016). VSEARCH: A versatile open source tool for metagenomics. *PeerJ*, *4*, e2584. https://doi.org/10.7717/peerj.2584

Tanabe, A. S., & Toju, H. (2016). Correction: Two New Computational Methods for Universal DNA Barcoding: A Benchmark Using Barcode Sequences of Bacteria, Archaea, Animals, Fungi, and Land Plants. *PLOS ONE*, *11*(3), e0152242. https://doi.org/10.1371/journal.pone.0152242

Toju, H., Vannette, R. L., Gauthier, M.-P. L., Dhami, M. K., & Fukami, T. (2018). Priority effects can persist across floral generations in nectar microbial metacommunities. *Oikos*, *127*(3), 345–352. https://doi.org/10.1111/oik.04243

Zhang, J., Kobert, K., Flouri, T., & Stamatakis, A. (2014). PEAR: A fast and accurate Illumina Paired-End reAd mergeR. *Bioinformatics*, *30*(5), 614–620. https://doi.org/10.1093/bioinformatics/btt593
